# Erlotinib can halt adenine induced nephrotoxicity in mice through modulating ERK1/2, STAT3, p53 and apoptotic pathways

**DOI:** 10.1038/s41598-020-68480-7

**Published:** 2020-07-13

**Authors:** Ahmed M. Awad, Mohamed A. Saleh, Nashwa M. Abu-Elsaad, Tarek M. Ibrahim

**Affiliations:** 1 Pharmacology and Toxicology Department, Faculty of Pharmacy, Mansoura University, El Gomhoria Street, Mansoura, Eldakahlia 35516 Egypt; 2 Department of Clinical Sciences, College of Medicine, University of Sharjah, Sharjah, United Arab Emirates

**Keywords:** Nephrology, Kidney diseases, Experimental models of disease

## Abstract

Renal fibrosis is a failed regenerative process that facilitates chronic kidney disease progression. The current study was designed to study the effect of erlotinib, a receptor tyrosine kinase inhibitor, on the progression of renal fibrosis. The study included four groups of mice: control group; adenine group: received adenine (0.2% w/w) daily with food for 4 weeks; erlotinib group: received 80 mg/kg/day erlotinib orally (6 ml/kg/day, 1.3% w/v suspension in normal saline 0.9%) for 4 weeks; adenine + erlotinib group: received adenine and erlotinib concurrently. Kidney function and antioxidant biomarkers were measured. Renal expression of Bcl2 and p53 and histopathological changes (tubular injury and renal fibrosis) were scored. Renal tissue levels of transforming growth factor-β_1_, p-ERK1/2 and p-STAT3 were measured. Results obtained showed significant decrease (*P* < 0.001) in serum creatinine, urea and uric acid in erlotinib + adenine group. Level of malondialdehyde was decreased significantly (*P* < 0.001) while reduced glutathione and catalase levels were increased (*P* < 0.01) by erlotinib concurrent administration. Erlotinib markedly reduced fibrosis and tubular injury and decreased TGF-β1, p-ERK1/2 and p-STAT3 (*P* < 0.5). In addition, expression level of Bcl-2 was elevated (*P* < 0.001) while that of p53-was reduced compared to adenine alone. Erlotinib can attenuate renal fibrosis development and progression through anti-fibrotic, antioxidant and anti-apoptotic pathways.

## Introduction

Chronic kidney disease (CKD) is a global health burden and is considered as an independent risk factor for cardiovascular diseases**.** Its prevalence is correlated to many health problems as diabetes, hypertension and obesity^1, 2^.

Renal fibrosis is a failed regenerative process that facilitates the development of CKD. The progression of renal insufficiency in patients with CKD is mainly due to tubulointerstitial fibrosis, glomerular inflammation and tubular atrophy^3^. Many cellular events are involved in tubulointerstitial fibrosis as infiltration of inflammatory cells, fibroblast activation, extracellular matrix (ECM) deposition and microvascular rarefaction^4^.

Various mediators are involved in the development of CKD and may represent possible targets for therapy such as transforming growth factor (TGF)-β, nuclear factor-erythroid 2 related factor, peroxisome proliferator-activated γ receptor, IL-11 and advanced glycation endproducts^5, 6^. Recently, studies reported involvement of miRNA in the progression of CKD progression^7^.

Tyrosine kinases have a pivotal role in cell signalling, and their activity deregulation can promote the development and progression of fibrotic diseases. As a result, pathologic activation of tyrosine kinases can drive fibrogenesis^8^.

Epidermal growth factor receptor (EGFR), a receptor tyrosine kinase, has been identified in renal tissue mainly in the epithelial cells of distal and collecting tubules, peritubular vessels and glomeruli with more abundant expression in diseased than in normal kidneys^9, 10^. Activation of these receptors has been found to contribute in various experimental models of CKD as diabetic nephropathy and glomerulonephritis^11–13^. Recently, targeting EGFR by blocking and/or interfering with its signalling pathway showed a protective effect against renal fibrosis^14, 15^.

Erlotinib is an EGFR tyrosine kinase selective reversible inhibitor that competes with the binding of ATP to the receptor intracellular domain. It thereby inhibits receptor autophosphorylation and blocks downstream signals^16^. Erlotinib is used in treatment of different types of cancer as lung cancer^17^ and head and neck squamous cell carcinoma^18^. Many studies showed beneficial effects of erlotinib in different kidney injuries. Matar et al. (2013) demonstrated that erlotinib can prevent salt retention and partially preserve renal function in nephrotic rats^19^. It can also suppress progression of glomerulonephritis through attenuating ultrastructural alterations of podocytes^20^. Yamamoto et al. (2018) revealed that erlotinib can attenuate progression of CKD in 5/6Nx rats^21^.

Collectively, EGFR signaling presents an interesting therapeutic target in the treatment of fibrotic renal disease. The present study was carried out to investigate effect of erlotinib as an EGFR inhibitor in an experimental model of chemically induced nephrotoxicity and fibrosis.

## Results

### Kidney function biomarkers, tubular injury and fibrosis

**Figure 1:**
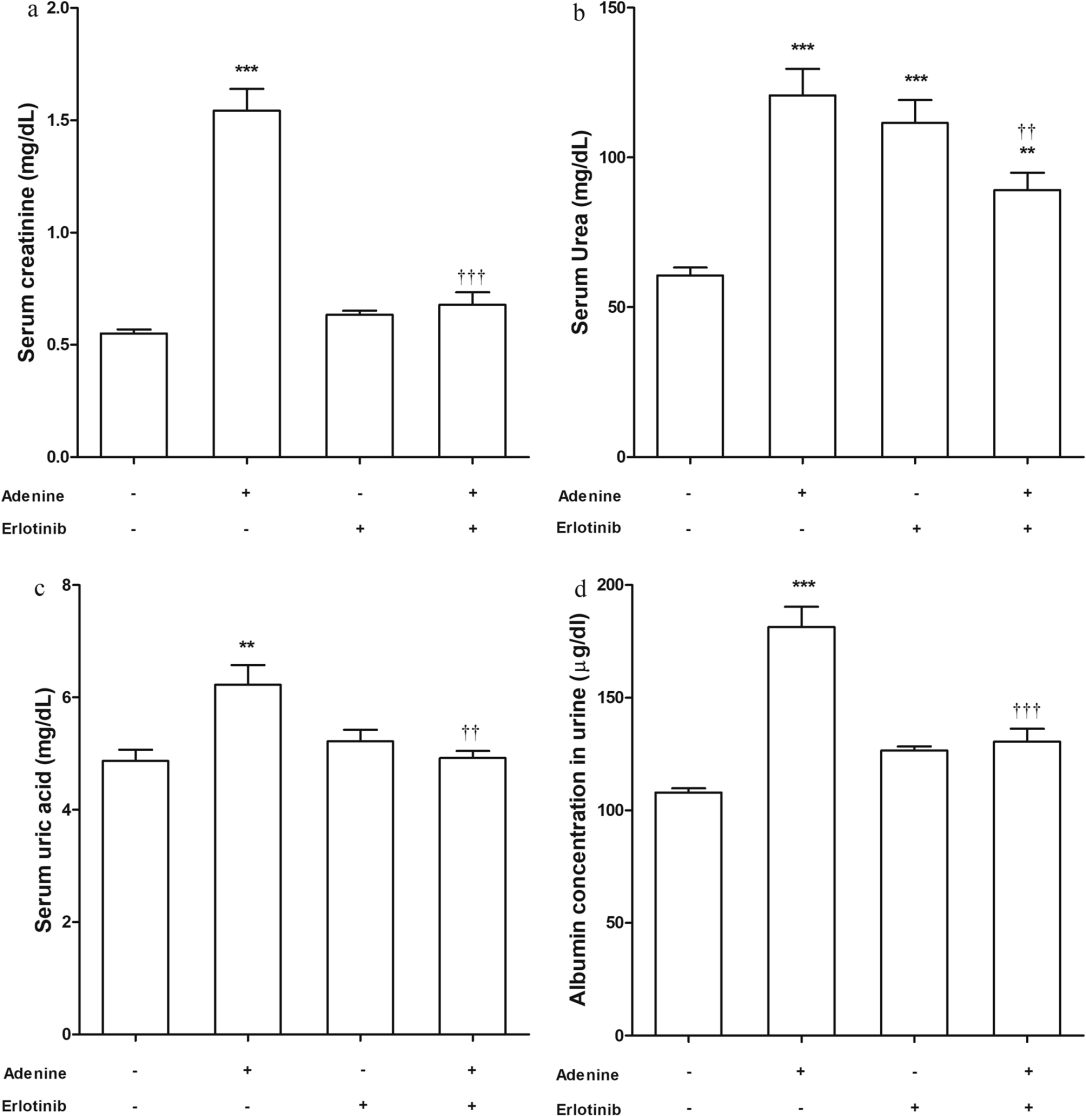
Effect of erlotinib (80 mg/kg) administration on (**a**) serum creatinine, (**b**) serum urea, (**c**) serum uric acid and (**d**) urine albumin levels in adenine induced nephrotoxicity in mice. Significance: **, *** *P* < 0.01, 0.001 compared to control group; ^‡‡^, ^‡‡‡^ *P* < 0.01, 0.001 compared to adenine group.

**Figure 2:**
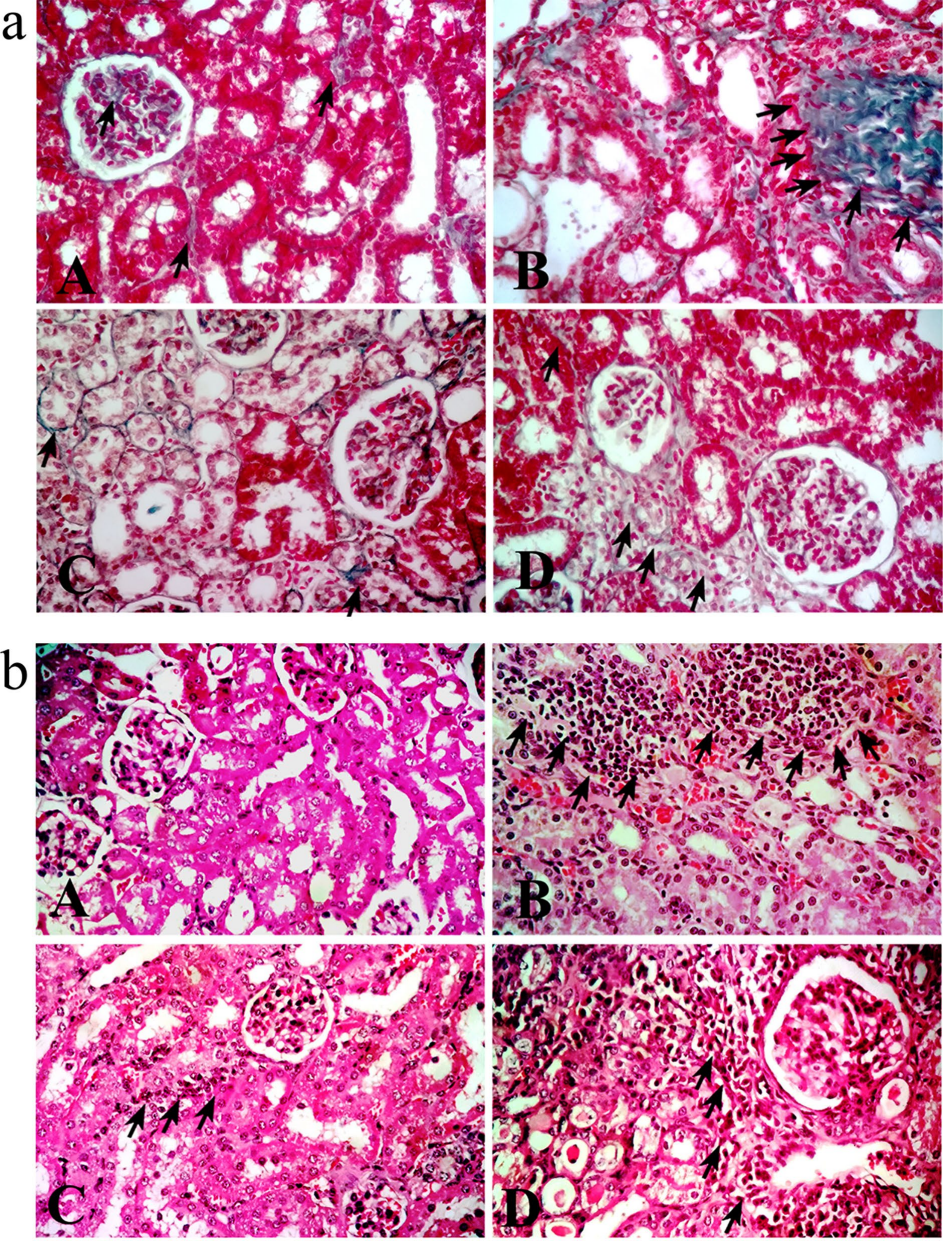
Representative photographs for kidney sections stained with a. Masson trichrome stain (× 400) showing fibrotic areas (arrows) (**b**). Hematoxylin–eosin stain (× 400) showing tubular injury and infiltration of inflammatory cells (arrows). A: control group B: adenine group C: erlotinib group D: adenine + erlotinib group.

Administration of adenine orally with food daily for 4 weeks resulted in significant elevation in serum creatinine, urea (*P* < 0.001) and uric acid (*P* < 0.01) levels compared to control group (Fig. 1a, b, c respectively). Also, there was a significant high albumin level in urine collected from this group (*P* < 0.001) (Fig. 1d). In line with these results, histopathological examination revealed marked tubular injury and significant fibrosis in adenine received group (Fig. 2a). Kidney sections isolated from adenine received mice were infiltered with remarkable mixed inflammatory cells (macrophages and neutrophils) indicating inflammation (Fig. 2b).

Administration of erlotinib concurrently with adenine resulted in significant (*P* < 0.01) decrease in levels of kidney function biomarkers compared to their elevated levels in group received adenine alone. The measured biomarkers (albumin in urine, serum creatinine and uric acid) in adenine + erlotinib group were comparable to control group level but urea level remained significantly high compared to control group level (*P* < 0.01).

Administration of erlotinib with adenine significantly decreased tubular injury score by about 1.5-fold when compared to adenine group score. Besides, it significantly decreased fibrosis score by about 2.4-fold when compared to fibrosis score in adenine group (Table 1).

**Table 1:** Effect of erlotinib (80 mg/kg) on renal tubular injury and fibrosis score in adenine induced renal injury in mice.

	Tubular injury^1^	Fibrosis score^1^
Control	0.00 ± 0.000	1.13 ± 0.034
Adenine	2.35 ± 0.150	3.32 ± 0.094***
Erlotinib	1.40 ± 0.112	1.37 ± 0.059
Adenine + erlotinib	1.60 ± 0.112^**‡‡**^	1.36 ± 0.050^**‡‡‡**^

^1^Scores were calculated /20 fields.

*** *P* < 0.001 compared to control group.

^**‡‡**^, ^**‡‡‡**^ *P* < 0.01, 0.001respectively compared to adenine group.

### Lipid peroxidation and antioxidant biomarkers

**Figure 3:**
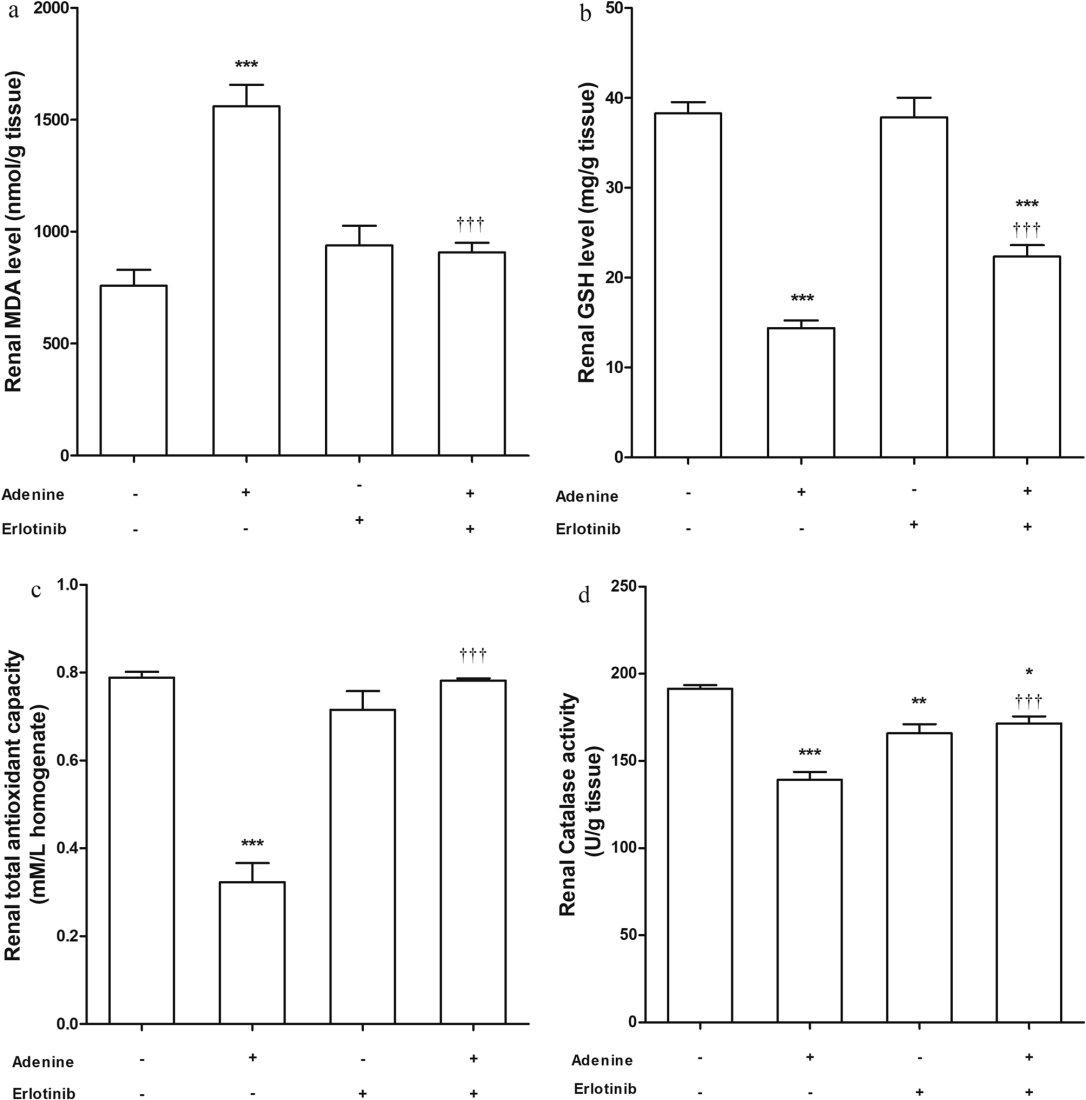
Effect of erlotinib (80 mg/kg) administration on renal (**a**) malondialdehyde (MDA) level, (**b**) reduced glutathione (GSH) level, (**c**) total antioxidant capacity and (**d**) catalase activity in adenine induced nephrotoxicity in mice. Significance: *, **, *** *P* < 0.05, 0.01, 0.001 compared to control group; ^‡‡‡^ *P* < 0.001 compared to adenine group.

Adenine administration increased level of malondialdehyde (MDA) significantly (*P* < 0.001) while the group received erlotinib concurrently with adenine showed a decreased (*P* < 0.001) MDA level compared to adenine group (Fig. 3a). Reduced glutathione (GSH) level in kidney homogenate (Fig. 3b) was significantly high (*P* < 0.001) in adenine + erlotinib group compared with its level in adenine group. Notably, GSH level in the former group was still lower than the control group level (*P* < 0.001).

Erlotinib significantly increased (*P* < 0.001) total antioxidant capacity when administered with adenine compared to adenine alone (Fig. 3c). Catalase level in renal tissue was significantly low in groups received adenine alone (*P* < 0.001) and erlotinib alone (*P* < 0.01). Concurrent administration of both adenine and erlotinib significantly elevated catalase level (*P* < 0.001) but remained significantly lower than control group level (*P* < 0.05) (Fig. 3d).

### Profibrogenic marker TGF-β_1_, p-ERK1/2 and phospho-STAT3

**Figure 4:**
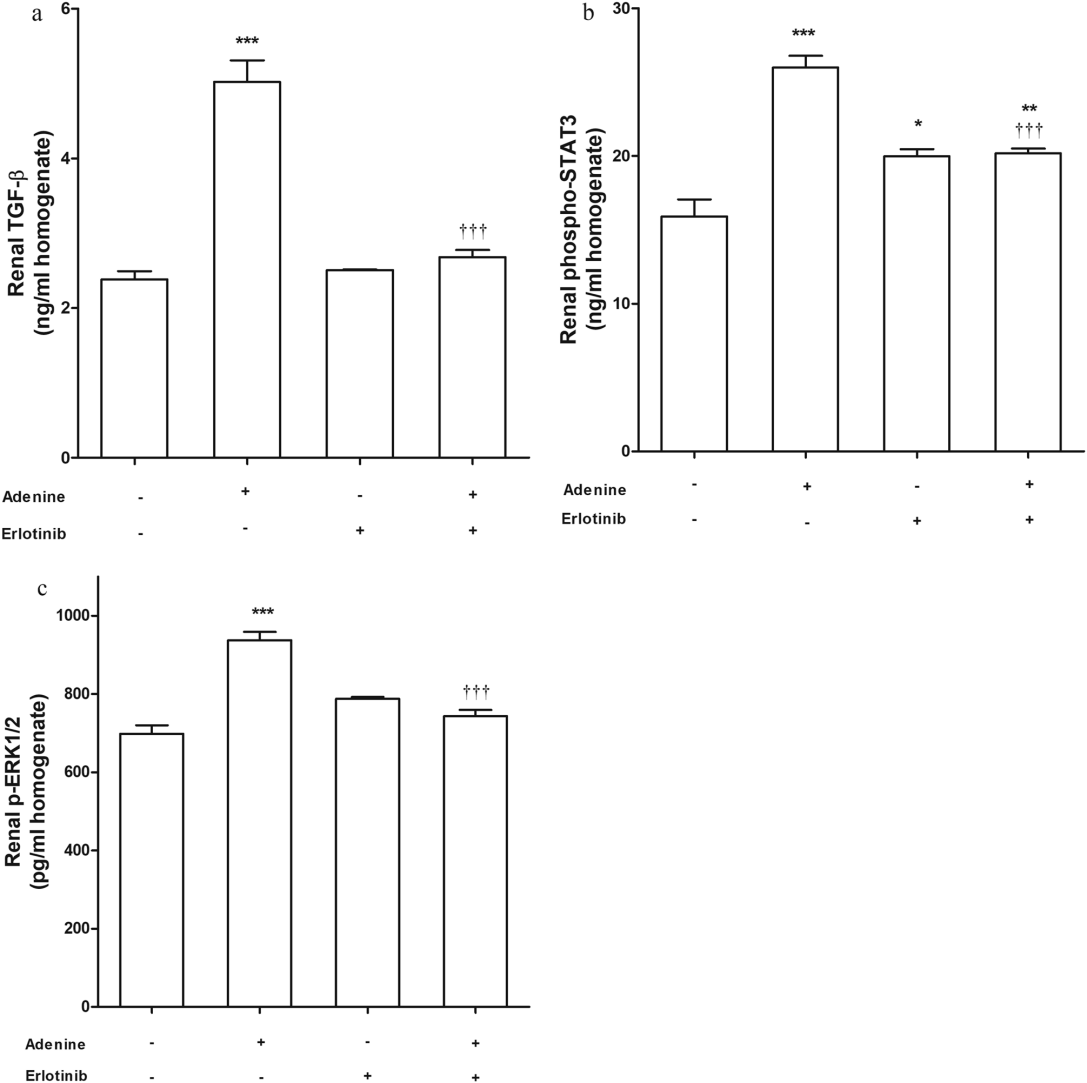
Effect of erlotinib (80 mg/kg) administration on renal levels of (**a**) transforming growth factor (TGF)-β_1_, (**b**) phospho-signal transducer and activator of transcription (STAT)-3, (**c**) p-extracellular signal-regulated kinase (ERK)-1/2 in adenine induced nephrotoxicity in mice. Significance: *, **, *** *P* < 0.05, 0.01, 0.001 compared to control group; ^‡‡‡^ *P* < 0.001 compared to adenine group.

Measuring level of TGF-β_1_ in kidney tissue showed an elevated level of the fibrogenic marker significantly (*P* < 0.001) in adenine group compared to control group (Fig. 4a). In adenine + erlotinib group, TGF-β_1_ level was significantly (*P* < 0.001) less than its level in adenine group. Similar results were obtained when measuring kidney levels of phosphorylated extracellular signal-regulated kinases-1 and 2 (p-ERK1/2) [Fig. 4b].

Renal phosphorylated signal transducer and activator of transcription-3 (phospho-STAT3) level (Fig. 4c) was significantly (*P* < 0.001) high in adenine received group compared to its level in control group. The group received erlotinib alone also showed a significant (*P* < 0.05) high level of phospho-STAT3. Erlotinib administration with adenine significantly lowered (*P* < 0.001) phospho-STAT3 level compared to adenine group level. Phospho-STAT3 level in adenine + erlotinib group was significantly higher than control group level (*P* < 0.01).

### Expression of Bcl-2 and p-53

**Figure 5:**
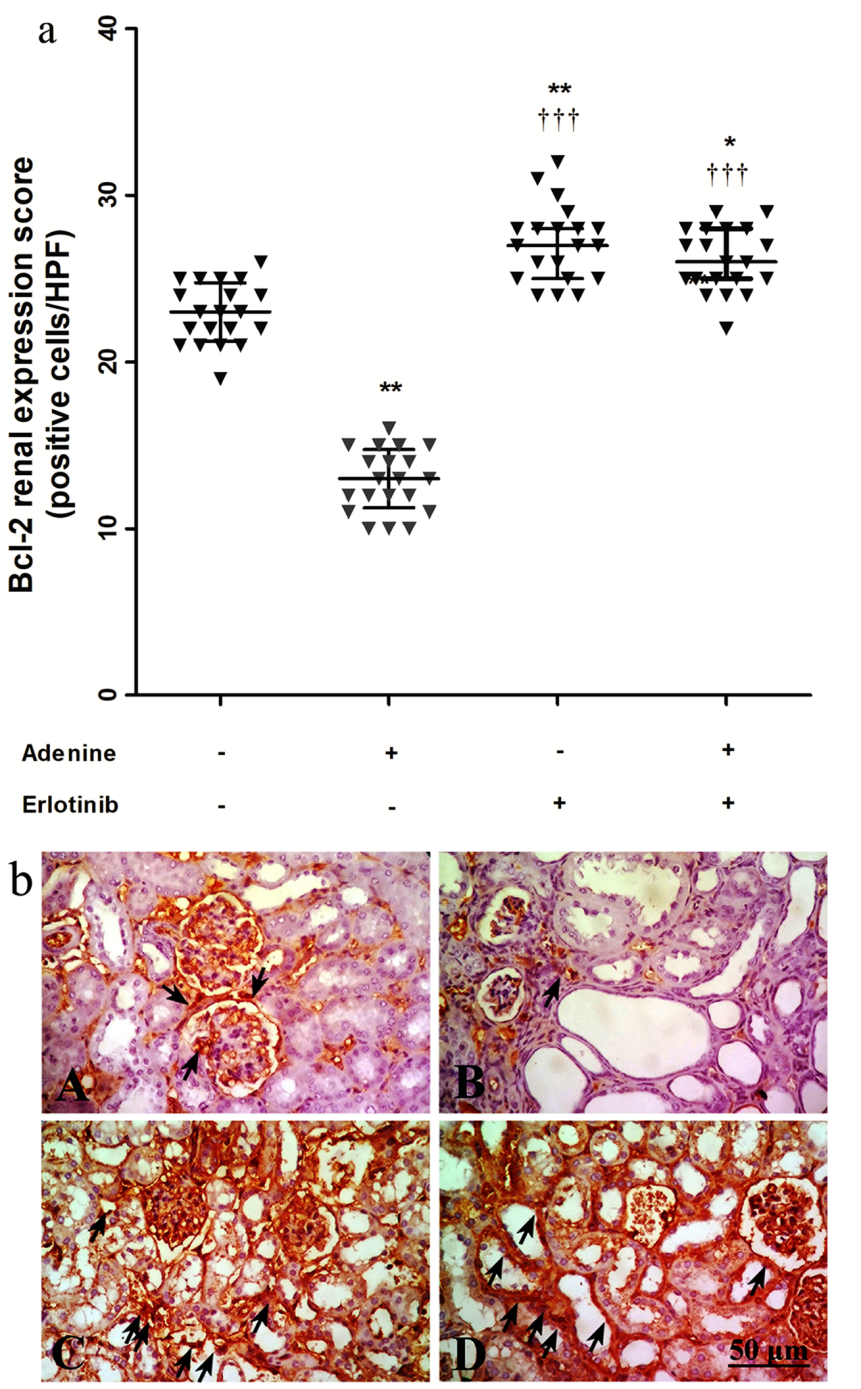
(**a**) Effect of erlotinib (80 mg/kg) administration on kidney expression scores of B-cell lymphoma (Bcl)-2 in adenine induced nephrotoxicity in mice. Significance: *, ** *P* < 0.05, 0.01 compared to control group; ^‡‡‡^ *P* < 0.001 compared to adenine group. (**b**) Representative photographs for immuno-stained kidney sections isolated from A: control group B: adenine group C: erlotinib group D: adenine + erlotinib group showing expression of Bcl-2 (arrows).

Figure 5a shows expression scores of B-cell lymphoma (Bcl)-2 in immuno-stained kidney sections (Fig. 5b). Adenine administration resulted in 1.8-fold decrease in Bcl-2 expression compared to control group. Administration of erlotinib concurrently with adenine significantly increased Bcl-2 expression (*P* < 0.001) compared to adenine alone. Expression scores in erlotinib and adenine + erlotinib groups (*P* < 0.01, 0.05 respectively) were significantly higher than control group score.

**Figure 6:**
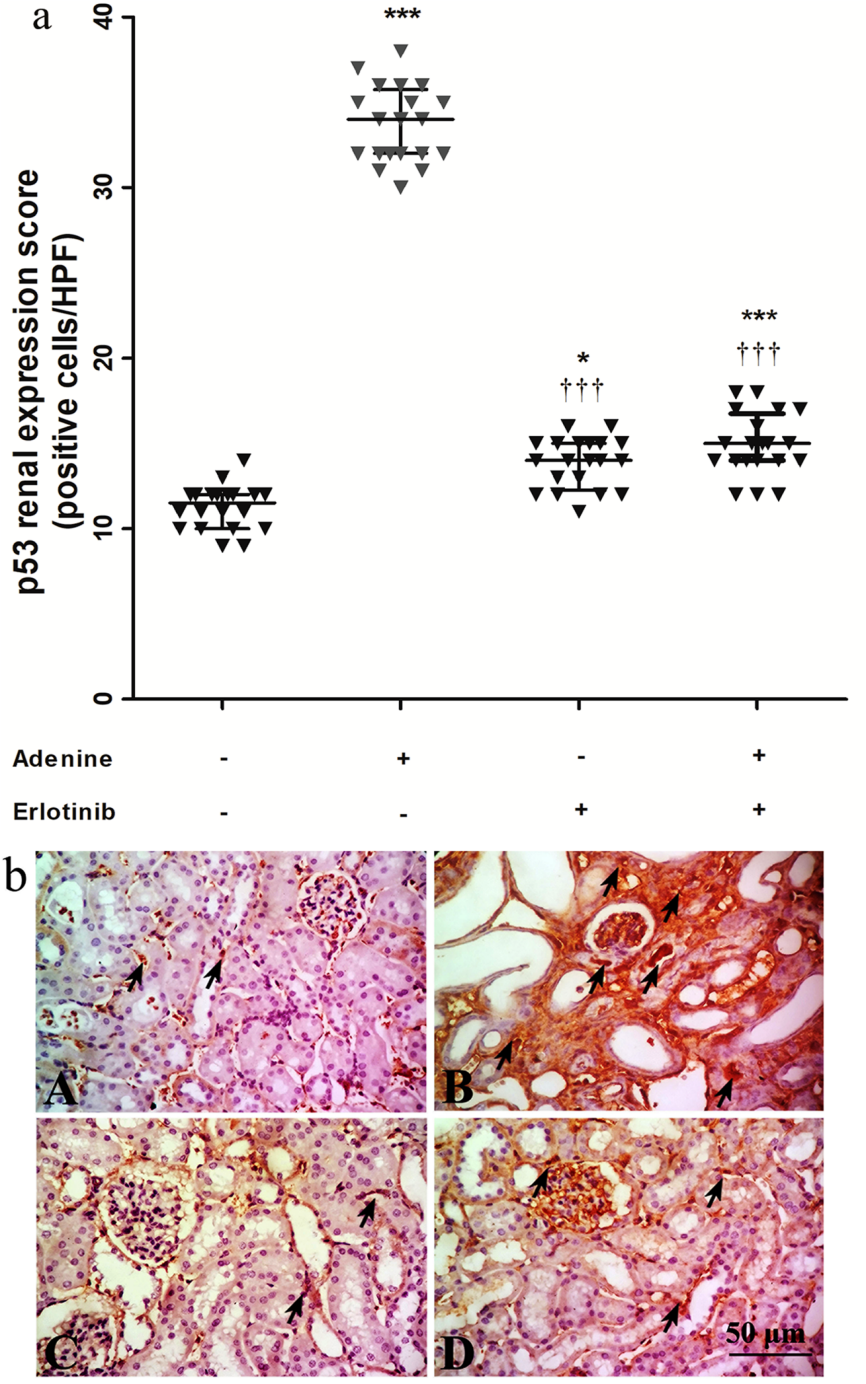
(**a**) Effect of erlotinib (80 mg/kg) administration on kidney expression scores of tumor protein p-53 in adenine induced nephrotoxicity in mice. Significance: *, *** *P* < 0.05, 0.001 compared to control group; ^‡‡‡^ *P* < 0.001 compared to adenine group. (**b**) Representative photographs for immuno-stained kidney sections isolated from A: control group B: adenine group C: erlotinib group D: adenine + erlotinib group showing expression of p-53 (arrows).

On the other hand, expression of the tumor protein p-53 in adenine group was markedly increased by ≈ 3-fold compared to its expression in control group (Fig. 6a). Expression was significantly decreased in erlotinib and adenine + erlotinib groups (*P* < 0.001) compared to adenine group but still significantly (*P* < 0.05, 0.001 respectively) higher than control group. Immuno-stained sections representing p-53 expression is shown in Fig. 6b.

## Discussion

Renal fibrosis has been reported to be a final common pathway in progressive renal diseases that subsequently leads to end-stage renal disease^20^. Currently, no effective treatment can reverse or even halt the progression of CKD and therapies that target fibrosis underlying mechanisms remains a challenge.

The current study was designed to investigate the effect of erlotinib as an EGFR blocker in protection against adenine induced renal fibrosis in mice. Effect of erlotinib on kidney function biomarkers, oxidative stress and antioxidant biomarkers was evaluated. In addition, renal sections were examined for inflammatory cells infiltration, tubular injury and collagen deposition. Effect on fibrogenic pathways was assessed by measuring TGF-β_1_ level. We also evaluated effect of erlotinib on expression of p53, Bcl2, phosphor-STAT3 and p-ERK1/2 to track possible mechanism underlying its renoprotective effect.

Dietary adenine has been extensively used to induce progressive kidney injury that highly mimic human CKD. Adenine model was established mainly in mice or rats and was used to test therapeutic strategies that may slow progression or even reverse renal injury and fibrosis^22^. After oral administration, adenine metabolites precipitates as crystals in renal tubules leading to tubulointerstitial nephritis and vascular calcification as well^23^.

To our knowledge there is no reported data that signify harmful effects of erlotinib on kidney function. In addition, it has been used safely as an anticancer for small cell lung cancer in hemodialysis patients^24^. Otherwise, concerns about liver complications has been raised with erlotinib use in treating pancreatic cancer and liver transaminases monitoring was recommended^25^.

Obtained results showed an elevation in serum urea level in erlotinib group suggesting a metabolic impact of erlotinib as a tyrosine kinase inhibitor on liver cells. Previous studies on cancer cell lines showed an effect of tyrosine kinase inhibitors on N-acetylglutamate synthase a key enzyme in the urea cycle^26, 27^. We also postulate that erlotinib may interfere with glutamate incorporation in ERK1/2 stimulatory signals through halting glutamate receptors activation by Src tyrosine kinases^28, 29^. This effect may in turn direct glutamate towards urea formation elevating its blood level.

EGF/EGFR system is involved in several biological processes including proliferation, differentiation and survival. It activates proliferation of tubular cells in autocrine/paracrine manner^30^. The glomerulus and the tubulointerstitial compartment show specific localization of EGFR^10^ and interestingly, upregulation of the EGFR and their pathway activation was reported in both human and experimental chronic renal diseases^31^.

Various publications postulate a key role for EGFR pathway in the pathogenesis of rapidly progressive glomerulonephritis and other forms of glomerulosclerosis, diabetic nephropathy and chronic allograft nephropathy^32^. In the present study, the elevated levels of serum creatinine, uric acid and urine albumin in adenine group were decreased by concurrent erlotinib administration. This is consistent with the effect of targeting EGF/EGFR by erlotinib in protection against glomerular injury and improvement of glomerular filtration in nephrectomy model^21^.

The kidney is a highly energetic organ and so it is liable to oxidative stress induced damage. In turn, oxidative stress can enhance renal disease progression^33^. Studies reported a link between EGF-EGFR and reactive oxygen species (ROS) production. For example, a cross-talk between EGF-EGFR axis and NADPH oxidase mediated ROS signaling pathways was reported. The enzyme NADPH oxidase is considered a major source of ROS in many cell types such as endothelial and smooth muscle cells^34, 35^. Additionally, EGF addition to human epidermoid carcinoma A431 cells, which highly express the EGFR, resulted in increased levels of intracellular ROS^36^. Furthermore, a key role of EGFR/AKT/ROS stress signaling in the development of nephropathy has been proved^37^.

Administration of adenine in our study resulted in high levels of MDA and a reduction in endogenous antioxidant biomarkers. Elevation of renal MDA level was observed in different experimental models of renal fibrosis as unilateral ureteral obstruction and lipopolysaccharide induced renal fibrosis^38, 39^. Malondialdehyde is an indirect marker of lipid peroxidation. High level of MDA indicates ROS production that initiates tissue injury as an early leading step to fibrosis. In line with previous researches, data obtained suggests a renoprotective effect of erlotinib through an antioxidant capability. It reduces MDA level and promotes different antioxidant pathways such as GSH production, catalase activation and increasing total antioxidant capacity.

In renal injuries, the kidney activates various signaling networks as those containing mitogen-activated protein kinases (MAPKs)^40, 41^ including ERK1/2 that is mainly activated by mitogenic stimuli such as growth factors and hormones^42^. Robust ERK activation has been reported in many kidney diseases as polycystic kidney disease^43^ glomerulonephritis^44^ and many others. Nowak (2002) suggested a role of ERK1/2 inhibition in attenuating cisplatin induced renal apoptosis and tissue injury^45^. Moreover, targeting EGFR/ERK1/2 pathway using inhibitors showed slow renal fibrosis progression in different models of fibrosis^46–48^.

In various experimental and human nephropathies, STAT3 is activated in many compartments of the injured kidney^49, 50^. It contributes to fibrosis by inducing collagen type 1 synthesis with subsequent increased ECM deposition. Kidney fibrosis models attribute TGF-β_1_ increased level and the subsequent collagen I production to STAT3^51^. Interestingly, both STAT3 pharmacologic inhibition and/or haploinsufficiency have been reported to decrease injury progression in different types of nephropathy^52, 53^. Our study showed ability of erlotinib to inhibit STAT3 and ERK1/2 phosphorylation activated by adenine and as a result renal injury and fibrosis are attenuated.

Being an important fibrogenic cytokine, TGF-β_1_ induces production of ECM and alters its degradation and involved in the major pathogenic mechanisms of CKD. In models of renal fibrosis, activation of EGFR signaling was critical for increasing production of TGF-β_1_^52, 54^. Overexpression of TGF-β_1_ was found to be mediated through ERK1/2 downstream of EGFR in vitro^55^. Chen et al. (2011) reported that inhibition of EGFR can attenuate TGF-β_1_ induced cell mitogenesis via the EGFR-ERK1/2/p38 kinase pathway in cultured renal interstitial fibroblasts^56^. In line with these findings, erlotinib herein decreased the profibrogenic marker TGF-β_1_ an effect that may be mediated by halting the activation of ERK1/2 pathway.

Apoptosis is a cell-suicide program that occurs through either death receptor-mediated extrinsic pathway and/or the mitochondria-mediated intrinsic pathway^57^. The mitochondrial pathway involves the key regulators Bcl-2 family proteins. The balance between the anti-apoptotic member Bcl-2 and the pro-apoptotic member Bax can regulate the activation of caspases. Recent studies reported the downregulation of Bcl-2 in renal tissue and activation of caspase pathway in chronic renal failure^58^.

Likewise, our results showed that the expression of Bcl-2 was markedly decreased in mice received adenine whereas treatment with erlotinib increased its renal expression suggesting anti-apoptotic effect of erlotinib and so protection against renal tissue damage.

Moreover, activation of the tumor suppressor p53 in tubular cells also play a role in acute kidney injury initiation and maladaptive kidney repair. Inhibition of p53 could be a novel and potential strategy for the treatment acute injury and its progression into CKD^59^. Samarakoon et al. (2013) suggested that p53 is profibrotic effector required for expression of a subset of profibrotic genes in response to TGF-β_1_^60^. They demonstrated cooperativity between p53, SMAD3 and EGFR signaling in inducing the profibrotic gene expression. Accordingly, erlotinib ability to regress renal fibrosis in the current study may be, at least in part, attributed to the reduction in p-53 expression in kidney tissue.

## Conclusion

**Figure 7:**
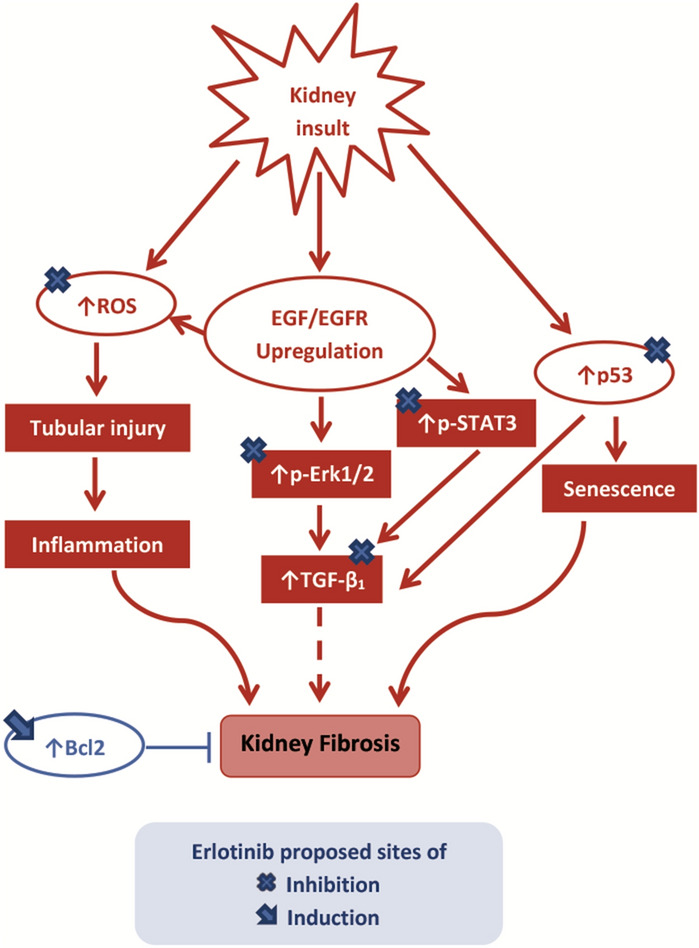
Graphical abstract. *Bcl* B-cell lymphoma, *EGF* epidermal growth factor, *EGFR* epidermal growth factor receptor, *p-STAT3* phosphorylated signal transducer and activator of transcription-3, *p-ERK* phosphorylated extracellular signal-regulated kinase, *ROS* reactive oxygen species, *TGF* transforming growth factor.

The EGFR inhibitor erlotinib can attenuate progression of kidney injury and renal fibrosis. This effect may be mediated at least by antioxidant effect, targeting STAT3 activation, ERK1/2 pathway and the profibrogenic marker TGF-β_1_. Interestingly, erlotinib was found to modulate apoptosis through up-regulating the anti-apoptotic member Bcl-2. Finally, it interferes with p-53 induced fibrotic genes. Since erlotinib was administered concurrently with adenine and showed an ability to slow fibrosis progression, future research about its ability to reverse fibrosis is warranted. Figure 7 summarizes proposed mechanisms underlying erlotinib renoprotective effect.

## Materials

*Animals*: Albino mice (n = 40, 25–30 g, adult male, *Swiss CD-1* strain) were purchased from VACSERA [Agouza, Giza, Egypt] and housed 10 mice/cage randomly. During accommodation period of 2 weeks mice were allowed food and water ad libitum. The experimental protocol approved by the Scientific Research Ethics Committee (Faculty of Pharmacy, Mansoura University) for the care and use of laboratory animals under approval number 2019-151. The study was carried out according to the guidelines and ethical principles adopted by the mentioned committee.

*Chemicals*: Adenine (99%) as fine white powder (Oxford laboratory Pvt. Ltd., Mumbai, India; PubChem CID: 190); Erlotinib free base as white powder (LC laboratories, MA, USA; PubChem CID: 176870).

### Induction of renal fibrosis

Mice average food consumption in each cage was reported during accommodation. Each cage was found to consume about 50 g food/day. Food was restricted to this average in all groups during the study. Renal fibrosis was induced in mice by receiving adenine with food (0.2% w/w) daily for 4 weeks as previously described by Nemmar et al.^61^.

### Experimental design

Four groups of mice (n = 10 each) were used as follows: control group: received erlotinib vehicle (6 ml/kg/day normal saline orally using oral gavage for 4 weeks); adenine group: received adenine (0.2% w/w) daily with food four 4 weeks in addition to erlotinib vehicle as described in control group; erlotinib group: received 80 mg/kg/day erlotinib^62^ orally (6 ml/kg/day, 1.3% w/v suspension in normal saline 0.9%) for 4 weeks; adenine + erlotinib group: received adenine and erlotinib concurrently as described above. Schematic presentation of experimental design is shown in Table 2.

**Table 2:** Schematic presentation of experimental design.

Group	Week				
	1	2	3	4	
Control	○	○	○	○	Sacrifice
Adenine	■○	■○	■○	■○	
Erlotinib	●	●	●	●	
Adenine + erlotinib	■●	■●	■●	■●	

n = 10/group.

**○** Mice received 0.9% w/v saline (6 ml/kg/day, orally by oral gavage).

**■** Mice received 0.2% w/w adenine (orally/day with food).

**●** Mice received 80 mg/kg erlotinib (6 ml/kg/day, 1.3% w/v suspension in 0.9% w/v saline, orally by oral gavage).

## Methods

Twenty-four hours following the last adenine dose, mice were anesthetized (50 mg/kg thiopental, *i.p*), blood samples were collected through cardiac puncture and serum was separated by centrifugation (4000 rpm, 4 °C, 15 min). Kidneys were isolated, washed with saline and cut into pieces longitudinally. To prepare kidney homogenate, kidney pieces were minced manually PBS (10% w/v) using a handheld homogenizer on ice, centrifuged (4000 rpm at 4 °C for 20 min) and the supernatant was separated. Other kidney pieces were fixed in neutral buffered formalin (8% v/v) for 24 h for preparation of paraffin blocks. Urine samples were collected 24 h before sacrifice through transferring mice to metabolic cages along 24 h.

### Kidney function and antioxidant biomarkers

Levels (mg/dl) of serum creatinine, urea and uric acid were measured spectrophotometrically as indicated by the manufacturer (Biomed Diagnostics, Egy-Chem, Egypt). Albumin level (μg/dl) in urine was measured using commercial kits (ABC Diagnostics, Egypt).

Level of MDA (nmol/g tissue) as a lipid peroxidation indirect marker was measured in kidney homogenate as described by Ohkawa et al.^63^. Total antioxidant capacity and GSH level were measured spectrophotometrically (Bio-diagnostic, Giza Egypt) at 505 and 405 nm respectively. Activity of catalase was determined as previously described by Aebi^64^.

### Measurement of renal phospho-STAT3, TGF-β_1_ and p-ERK1/2

Transforming growth factor- β_1_ (Cloud Clone Corp., USA), phospho-STAT3 and p-ERK1/2 (Assay Biotechnology Co., CA, USA) were measured in kidney homogenate using sandwich ELISA technique according to the manufacturer’s instructions.

### Histopathological examination and immunohistochemistry (IHC)

Kidney sections of 4 μm thickness were prepared from paraffin wax blocks, stained with hematoxylin–eosin (H&E) and examined for tubular injury using light microscope (× 400) according to Kinomura et al. (2008) modified scoring system^65^. Briefly, randomly selected 20 fields were quantified for: swelling, desquamation from the tubular basement membrane, necrosis and vacuolar degeneration using a score from 0 to 5 as follows: 0: normal; 1: < 20% of the tubules are involved; 2: 20–40%; 3: 40–60%; 4: 60–80%; and 5: 80–100%).

Other kidney sections were stained with Masson trichrome stain for interstitial fibrosis quantification as described by Yamate et al.^66^. Five random fields were selected and examined with the Image J 1.32 as a color image analyzer where fibrotic area was measured avoiding the blood vessels in the OSOM and cortico-medullary junction.

For IHC, kidney sections were deparaffinized with xylene, dehydrated in decreasing concentrations of ethanol. Sections were treated with 3% H_2_O_2_/methanol for 10 min to block endogenous peroxidase activity. For antigen retrieval, sections were processed in EDTA then pre-incubated with normal rabbit serum to block nonspecific binding of antibodies. Kidney sections were incubated overnight with rabbit polyclonal primary antibodies (dilution 1:50) against p53 (ab131442, Abcam) Bcl-2 (PA5-20068, Invitrogen, Thermo Fisher Scientific, CA) at 4 °C.

Sections were incubated with secondary antibodies as described by Kane and Greenhalgh^67^ after washing with phosphate buffer saline. Slides were then stained (with H&E as a counter stain) and visualized for antibody binding using horseradish peroxidase- diaminobenzidine detection system (GBI Labs, WA, USA). Using a light microscope (X100), expression of p53 (cytoplasmic and nuclear) and Bcl-2 (cytoplasmic) was detected as % of positive cells in 100 cells counted per each high definition field and calculated as the mean of 20 fields in each group^68^.

### Statistical testing

Data (n = 10) are expressed as mean ± S.E.M except IHC scores that are expressed as median with interquartile range. Level of α was set to 0.05 and obtained data were compared using one-way analysis of variance (ANOVA), followed by Tukey–Kramer multiple comparison test as the post-Hoc test. Tubular injury and IHC scores were analyzed with Kruskal Wallis test by rank followed by Dunn's multiple comparison test. Statistical analysis and figures construction were performed using GraphPad Prism V5.01 (GraphPad Software Inc, CA, USA).

## Data Availability

All data generated or analyzed during this study are included in this published article.
